# Predictors for Locoregional Recurrence for Clinical Stage III-N2 Non-small Cell Lung Cancer with Nodal Downstaging After Induction Chemotherapy and Surgery

**DOI:** 10.1245/s10434-012-2800-x

**Published:** 2012-12-20

**Authors:** Arya Amini, Feiran Lou, Arlene M. Correa, Randall Baldassarre, Andreas Rimner, James Huang, Jack A. Roth, Stephen G. Swisher, Ara A. Vaporciyan, Steven H. Lin

**Affiliations:** 1 Department of Radiation Oncology, Unit 97, The University of Texas MD Anderson Cancer Center, Houston, TX USA; 2 Department of Thoracic and Cardiovascular Surgery, The University of Texas MD Anderson Cancer Center, Houston, TX USA; 3 Department of Thoracic Surgery, Memorial Sloan Kettering Cancer Center, New York, NY USA; 4 Department of Radiation Oncology, Memorial Sloan Kettering Cancer Center, New York, NY USA; 5 University of California, Irvine School of Medicine, Irvine, CA USA; 6 University of California, San Diego School of Medicine, San Diego, CA USA

## Abstract

**Purpose:**

Pathologic downstaging following chemotherapy for stage III-N2 NSCLC is a well-known positive prognostic indicator. However, the predictive factors for locoregional recurrence (LRR) in these patients are largely unknown.

**Methods:**

Between 1998 and 2008, 153 patients with clinically or pathologically staged III-N2 NSCLC from two cancer centers in the United States were treated with induction chemotherapy and surgery. All had pathologic N0-1 disease, and none received postoperative radiotherapy. LRR were defined as recurrence at the surgical site, lymph nodes (levels 1–14 including supraclavicular), or both.

**Results:**

Median follow-up was 39.3 months. Pretreatment N2 status was confirmed pathologically (18.2 %) or by PET/CT (81.8 %). Overall, the 5-year LRR rate was 30.8 % (*n* = 38), with LRR being the first site of failure in 51 % (22/+99877943). Five-year overall survival for patients with LRR compared with those without was 21 versus 60.1 % (*p* < 0.001). Using multivariate analysis, significant predictors for LRR were pN1 disease at time of surgery (*p* < 0.001, HR 3.43, 95 % CI 1.80–6.56) and a trend for squamous histology (*p* = 0.072, HR 1.93, 95 % CI 0.94–3.98). Five-year LRR rate for pN1 versus pN0 disease was 62 versus 20 %. Neither single versus multistation N2 disease (*p* = 0.291) nor initial staging technique (*p* = 0.306) were predictors for LRR. N1 status also was predictive for higher distant recurrence (*p* = 0.021, HR 1.91, 95 % CI 1.1–3.3) but only trended for poorer survival (*p* = 0.123, HR 1.48, 95 % CI 0.9–2.44).

**Conclusions:**

LRR remains high in resected stage III-N2 NSCLC patients after induction chemotherapy and nodal downstaging, particularly in patients with persistent N1 disease.

**Electronic supplementary material:**

The online version of this article (doi:10.1245/s10434-012-2800-x) contains supplementary material, which is available to authorized users.

For patients with stage III NSCLC, multimodality therapy remains the standard of care. Approximately 10 % of all NSCLC cases present as stage IIIA-N2, and for these patients, disease control and overall survival (OS) continue to be poor, with 5-year survival rates of 23 %.^1^ Several randomized trials have demonstrated local recurrence rates of 11–34 % among patients with stage I–III disease after surgery alone,^2^^–^ 4 suggesting additional consolidative therapies may be needed to control local disease. Earlier studies looking at consolidative therapies in addition to surgery in patients with stage III-N2 found this population to be quite heterogenous, making it difficult to determine the best treatment approach for these patients.

Several randomized trials and meta-analysis have demonstrated a survival benefit with neoadjuvant chemotherapy with surgery versus surgery alone.^5^^–^ 8 Most of the benefit of induction chemotherapy is restricted to more locally advanced, stage II–III patients.^9^ On further analysis, several studies found that patients with mediastinal nodal response after induction chemotherapy have improved outcomes.^7^^,^ 10 ^,^ 11 In one trial,^7^ induction chemotherapy produced 3- and 5-year survival rates of 67.7 and 51.6 % for patients with disease downstaged to pN0 compared with 38.5 and 17.6 % for those with pN1-3 disease. A study by the Swiss SAKK Group^11^ concluded that patients with nodal downstaging to N0-1 after induction therapy had improved disease-free survival and OS (HR 0.26) compared with patients with mediastinal lymph node involvement. The median time to local relapse comparing persistent pN2 versus pN0-1 was 14.4 versus 43.8 months. In their most recent update, they reported 5-year locoregional failure (LRF) rates as high as 60 % in the entire study population, including those with or without nodal downstaging. In those patients with pathologic response to chemotherapy, there was a significant reduction in distant metastasis. Although several other trials have demonstrated the prognostic importance of mediastinal nodal downstaging or pathologic response to induction chemotherapy, local failure rates specifically for the nodal downstaged group are rarely reported, especially in those patients who did not receive postoperative radiotherapy (PORT).

In this study, we performed a retrospective analysis of the treatment outcomes of patients treated at two major cancer centers to determine predictors for locoregional recurrence for patients with clinical stage III-N2 disease who undergo nodal downstaging after induction chemotherapy at the time of surgery. We hypothesized that there are certain patient and tumor characteristics that would be predictive for higher local recurrence and thus may necessitate more aggressive local treatment, including PORT.

## Methods and Materials

### Patient Selection

Chart review of all patients treated with induction chemotherapy followed by surgery between 1998 through 2008 was performed at The University of Texas MD Anderson Cancer Center and Memorial Sloan Kettering Cancer Center. We excluded patients with tumors not of non-small cell origin, having persistent N2 disease at the time of surgery, and death within 1 month of surgery. We identified 179 patients with stage III NSCLC (N2) who fit the above criteria. At the time of surgery, all patients in the study had documented downstaged nodal disease, either to N0 or N1. In addition, 26 patients who received PORT after surgery were excluded from the analysis, largely due to the relatively small sample size and were biased for high-risk characteristics (positive margins, bulky tumor). Based on these guidelines, 153 patients were identified and combined for this analysis: MD Anderson (*n* = 56) and Memorial Sloan Kettering (*n* = 97). This post hoc analysis was reviewed and approved by the institutional review boards of at both cancer centers.

### Treatment and Response Assessment

Both institutions followed the same guidelines in preparing and gathering patient and treatment characteristics. All patients had clinically or pathologically proven N2 involvement before starting induction chemotherapy. Clinically, patients were documented to have node-positive disease if the nodes were >1 cm in their shortest dimension, if the standardized uptake value (SUV) on positron emission tomography (PET) was >4.0, or both. The choice of neoadjuvant chemotherapy was at the discretion of the treating medical oncologist. All patients underwent surgical resection at MD Anderson or at Memorial Sloan Kettering Cancer Center. Tumor grade and histology were confirmed by pathological analysis at the time of surgery. Some patients received additional treatments, such as adjuvant chemotherapy but not PORT. The majority of patients (*n* = 131) received no additional chemotherapy after surgery. The Response Evaluation Criteria in Solid Tumors (RECIST) v1.1 was used to assess response to induction chemotherapy according to the change in the largest diameter of the tumor on PET/CT imaging.

### Local-Regional and Distant Recurrence

Local-regional recurrences were defined as those at the surgical site, at the anastomotic or bronchial stump, or in the local-regional lymph nodes (levels 1–14, including supraclavicular). Cervical and abdominal lymph node disease was considered distant recurrence. Disease recurrence was verified by either imaging (PET/CT) or biopsy. Time to recurrence was based on the date of imaging- or biopsy-proven disease recurrence and the original date of surgery.

### Follow-up and Survival

The date of diagnosis was based on the date of biopsy-proven NSCLC. This was compared with the last contact date, which was the last known date of the patients’ vital status and disease status, to calculate follow-up time. OS was calculated based on the initial date of diagnosis. A total of five patients had missing data on disease recurrence (either local-regional or distant or both) due to poor follow-up. Nine patients had an unknown vital status.

### Statistical Analysis

All statistical analyses were performed using the SPSS V17.0 (SPSS Inc., Chicago, IL). Pearson two-sided, chi-square tests were used to evaluate patient- and treatment-related differences between patients with or without local and distant recurrences. Univariate Cox regression analysis was performed using death, LRF, or DF as outcomes with a significance level of *p* < 0.05. Covariates that were significant at *p* < 0.25 were included in the multivariable Cox regression. Backward stepwise Wald elimination at *p* = 0.1 was used to establish the final model. Multivariate analysis was performed separately for overall survival, local, and distant metastasis. Patients with incomplete data were excluded from the multivariate analysis. Survival functions were calculated according to the Kaplan–Meier method, and differences were assessed with the log-rank test.

## Results

### Patient and Treatment Characteristics

From February 1998 to December 2008, 153 patients with clinically or pathologically staged III-N2 NSCLC from two cancer centers in the United States were treated with induction chemotherapy followed by surgery and found to have pathologically downstaged nodal disease at the time of their surgery. Median follow-up time for all patients was 39.3 (range 21.2–79) months. The 5-year overall survival for this population was 49.6 %. Patient characteristics are shown in Table 1. The median age was 65 (range 41–83) years; a slight majority were male (77.1 %), and most patients had good performance status (KPS ≥ 80). Nodal status before induction chemotherapy was staged by mediastinoscopy (15.7 %) or PET/CT (84.3 %). Most patients presented with T2 disease (63.4 %), single station N2 involvement (60.8 %), with adenocarcinoma being the most common histology found (54.2 %).

**Table 1:** Patient characteristics

Characteristics	No. of patients (%)
Age	
Median (range, years)	65 (41–83)
Gender	
Male	81 (52.9)
Female	72 (47.1)
Smoking status	
Never	10 (6.5)
Former	109 (71.2)
Current	34 (22.2)
Karnofsky performance status	
90–100	71 (46.4)
80	47 (30.7)
<80	11 (7.2)
Unknown	24 (15.7)
N2 status assessment method	
Mediastinoscopy	24 (15.7)
PET/CT	129 (84.3)
Clinical T status	
T1	38 (24.8)
T2	97 (63.4)
T3	16 (10.5)
T4	2 (1.3)
Level of N2 involvement at surgery	
Single station	93 (60.8)
Multistation	60 (39.2)
Tumor histology	
Adenocarcinoma	83 (54.2)
Squamous cell carcinoma	35 (22.9)
Other	35 (22.9)
Tumor grade	
Well	4 (2.6)
Moderate	46 (30.1)
Poor	78 (51.0)
Unclear	25 (16.3)
RECIST response	
CR/PR	72 (47.1)
SD/PD	81 (52.9)
Pathologic T status	
T0	10 (6.5)
T1	71 (46.4)
T2	48 (31.4)
T3	16 (10.5)
T4	8 (5.2)
Pathologic N status	
N0	117 (76.5)
N1	36 (23.5)
Lymphovascular invasion	
Yes	43 (28.1)
No	79 (51.6)
Unknown	31 (20.3)
Level of N2 involvement at surgery	
Single station	93 (60.8)
Multistation	60 (39.2)
Adjuvant chemotherapy	
Yes	22 (14.4)
No	131 (85.6)

*RECIST* response evaluation criteria in solid tumors

The median number of induction chemotherapy cycles was three (range 2–8), with 83 % of patients receiving a platinum–taxane doublet regimen. In this cohort, no patients received PORT; however, a small subset received adjuvant chemotherapy (*n* = 22, 14.4 %; Supplemental Table 1). The two most common reasons for receipt of systemic dose adjuvant chemotherapy were multilevel N2 disease, clinical T4 disease on initial presentation, or institutional protocols requiring additional adjuvant chemotherapy. Overall, 131 (85.6 %) patients did not receive postoperative chemotherapy.

### Local-Regional Failure Patterns

**Fig. 1:**
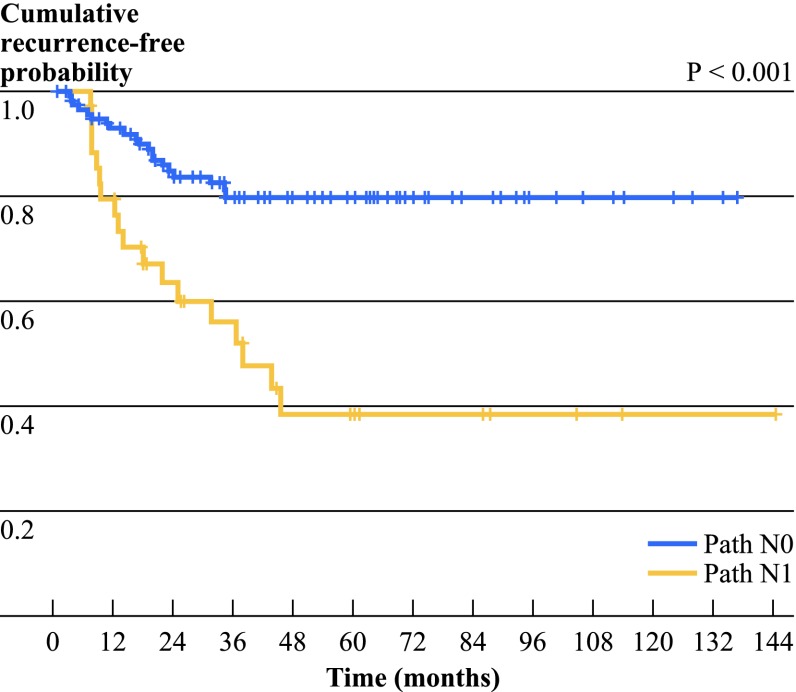
Kaplan–Meier curves illustrating cumulative locoregional (LR) recurrence free probability in pathologically downstaged N0 versus N1 disease

The 5-year local-regional failure (LRF) rate was 30.8 % (*n* = 38), with LRF being the first site of failure in 51 % of these individuals (22/43). All local recurrences in the study population occurred within 5 years, with the latest recurrence occurring 45.6 months after the date of surgery. On univariate analysis, LRF was significantly higher in patients with pathologic N1 compared with N0 disease (at time of surgery) (HR 3.478, 95 % CI 1.837–6.587, *p* *<* 0.001; Fig. 1). Additional predictors for LRF included squamous cell histology (HR 2.219, 95 % CI 1.086–4.535, *p* *=* 0.048). Neither single versus multistation N2 disease (HR 0.692, 95 % CI 0.349–1.371, *p* = 0.291) nor initial staging by mediastinoscopy versus PET/CT (HR 0.665, 95 % CI 0.305–1.451, *p* = 0.306) were predictors for LRF. Multivariate analysis demonstrated that only a pathologic N1 (at surgery) was associated with higher LRF rates (HR 3.435, 95 % CI 1.798–6.561, *p* *<* 0.001). There was only a trend for greater LRF rates in tumors with squamous cell histology (HR 1.934, 95 % CI 0.94–3.977, *p* *=* 0.073; Table 2).

**Table 2:** Multivariate analysis for locoregional failure

Covariate	Frequency	Hazard ratio (95 % CI)	*p* value
Tumor histology			
Adenocarcinoma	83	1.000 (NA)	0.072
Squamous cell	35	1.934 (0.94–3.977)	0.073
Other	34	0.726 (0.301–1.755)	0.477
Pathologic N status			
N1	36	3.435 (1.798–6.561)	<0.001
N0	116	1.000 (NA)	

### Distant Failure Patterns

**Fig. 2:**
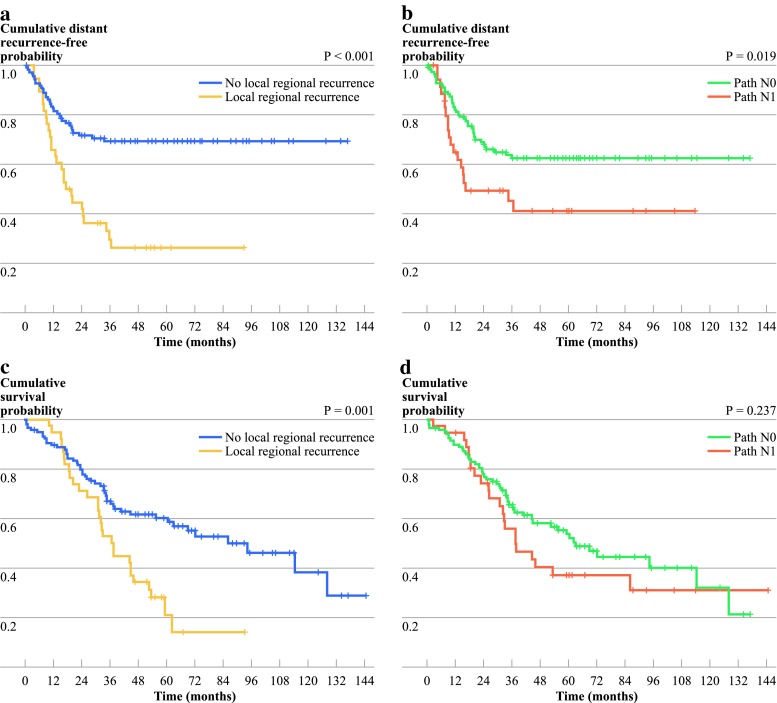
Kaplan–Meier curves illustrating cumulative distant metastatic-free survival in patients with or without locoregional recurrence (**a**) and in pathologically downstaged N0 versus N1 disease (**b**). Kaplan–Meier curves illustrating cumulative overall survival with or without locoregional recurrence (**c**) and in downstaged N0 versus N1 disease (**d**)

The probability of being distant recurrence-free at 5 years was 57.7 %; those with and without LRF had a 5-year distant recurrence-free disease of 26.3 and 69.3 %, respectively (*p* < 0.001; Fig. 2a). Under univariate analysis, the only predictor of higher distant failure (DF) rates was in those with pathologic N1 stage at the time of surgery (HR 1.909, 95 % CI 1.104–3.304, *p* = 0.021; Fig. 2b). In multivariate analyses, the pathologic N1 stage remained a significant independent predictor for higher distant failures (HR 1.909, 95 % CI 1.104–3.304, *p* = 0.021).

### Overall Survival

Five-year OS in our study cohort was 49.6 %; those with or without LRF had a 5-year OS of 21 % compared with 60.1 % (*p* = 0.001; Fig. 2c). Median survival for pN1 versus pN0 was 37.5 months compared with 62.8 months. Univariate analysis revealed that pathologic T4 disease (at surgery) was associated with a worse OS (HR 14.316, 95 % CI 1.717–119.327, *p* = 0.014). However, pathologic N0 versus N1 disease (at surgery) was not significantly associated with OS (HR 1.344, 95 % CI 0.821–2.2, *p* *=* 0.237; Fig. 2d). Higher pathologic T stage continued to be significant under multivariate analysis (Table 3).

**Table 3:** Multivariate analysis for overall survival

Covariate	Frequency	Hazard ratio (95 % CI)	*p* value
Age	153	1.023 (0.996–1.051)	0.091
Pathologic T status			
T0	10	1.000 (NA)	0.064
T1	71	5.934 (0.808–43.602)	0.08
T2	48	8.444 (1.15–61.981)	0.036
T3	16	8.624 (1.076–69.112)	0.042
T4	8	14.496 (1.738–120.87)	0.013

### Overall Recurrence Patterns

Most local regional recurrences appeared in the mediastinal (92.1 %) and hilar lymph nodes (23.7 %). Distant failures were the most common sites of failure in this group of patients (38.6 %). Most common site of distant metastasis was the brain (42.4 %) followed by the lung (22 %). Slightly more than half of the population had no disease relapse (51 %; Supplemental Table 2).

## Discussion

This study evaluated the rate of locoregional recurrence in patients with a pathologic response following neoadjuvant chemotherapy. Consolidative therapy in these patients is largely reserved for patients with bulky disease and positive margins at the time of surgery. Currently, adjuvant therapy for all patients with downstaged disease is not considered standard of care, even though local tumor failure rates continue to be high. In our study, we demonstrated a LRF rate of 30.8 %. In addition, those with pathologic N1 disease had significantly higher rates of LRF. For these patients, additional treatment following surgery may improve outcomes, because OS was significantly lower in patients with locoregional recurrence. Currently, the role for adjuvant therapy following surgery in nodal downstaged patients is largely unknown. Although PORT is more commonly prescribed to patients who are found to have residual N2 disease at the time of surgery, there is little agreement on additional radiation treatment for those with downstaged from N2 to N0-1 disease. One retrospective study looking at stage III-N2 disease treated with induction chemotherapy and surgery found no difference in outcomes between pN1 or pN2 at the time of surgery.^12^ Having any positive lymph nodes at the time of surgery was found to increase significantly the risk for recurrence (HR 1.9), suggesting those with pN1 to still be at high risk for recurrence.

Whereas numerous studies have shown evidence for improvement in local control with PORT, overall survival was not demonstrated to be better. The PORT meta-analysis compiling nine randomized trials from 1966 to 1994 demonstrated a worse overall survival (HR 1.21) in stage I–II NSCLC treated with PORT versus observation.^13^ However, the meta-analysis contained a heterogenous population from multiple institutions spanning a large time period, some of which used older and more toxic treatment techniques. More recent data using modern radiation fractionation, updated techniques, and equipment have supported the use of PORT, because the treatment-related mortality is much lower than expected from the older PORT trials.^14^^,^ 15

The need for adjuvant treatment in downstaged patients with proven N2 disease before induction chemotherapy continues to be discussed. There is presently little data on local control in patients with mediastinal downstaging following induction chemotherapy. Whereas prospective trials have shown mediastinal downstaging to be a positive prognostic indicator following induction chemotherapy, local failure rates in these patients are rarely analyzed. In one of the earlier induction chemotherapy randomized trials in stage III-N2 NSCLC, locoregional failure was seen in 54 % of recurrences.^5^ However, the relationship between local failures and pathologic response was not defined in the study. Taylor et al.^16^ conducted a retrospective analysis of patients with stage IIB–IIIA NSCLC treated with induction chemotherapy and found 5-year actuarial local control rates to be 82 % among patients with stage IIIA disease given PORT versus 35 % for patients with stage IIIA disease without PORT. No difference was observed in OS. However, this study did not report LRF rates according to extent of nodal downstaging. In the Swiss SAKK phase II trial,^17^ patients with stage III-N2 NSCLC received induction chemotherapy followed by surgery, but only patients with R1/R2 resections or upper mediastinal N2 disease received PORT. At 5 years, 60 % of patients had LRF and 65 % DF, again demonstrating high failure rates in such patients. The study however did not report failure patterns between pN2 versus pN0-1.

Our study is limited by its retrospective nature and that most of the disease recurrence determination was based on radiographic imaging with pathologic confirmation only attempted occasionally by biopsy. However, patients were started on palliative/salvage treatments based on these findings, with many of these patients to eventually further progress and die of their disease. Patients who were scored as having local recurrence had a poorer prognosis compared with patients without recurrence, verifying the validity of the initial call. The main strength of this study is that it is one of the first multi-institutional attempts to identify LRF rates and predictive factors in this specific cohort. The study also provided a detailed assessment of local-regional failure rates and patterns, which data often are missing from many induction chemotherapy studies.

In conclusion, the high local failure rates after nodal response in the mediastinum may suggest the need for additional consolidative treatment, such as PORT, specifically in patients at high risk for local recurrence. Patients who respond to induction chemotherapy may benefit the most from local treatment. As more effective systemic therapies are developed, there will be an even greater importance to control local disease, especially in those at high risk. Future clinical trials are needed to compare a variety of consolidative treatment options to define the best approach.

## Electronic supplementary material

Below is the link to the electronic supplementary material.

Supplementary material 1 (DOCX 13 kb)
