# Supplementary material for: Predictors for Locoregional Recurrence for Clinical Stage III-N2 Non-small Cell Lung Cancer with Nodal Downstaging After Induction Chemotherapy and Surgery
Source: Ann Surg Oncol. 2012 Dec 20;20(6):1934–40. doi: 10.1245/s10434-012-2800-x (PMC3656229; doi:10.1245/s10434-012-2800-x)
Supplement: Supplementary file 1 — Supplementary material 1 (DOCX 13 kb) [file 10434_2012_2800_MOESM1_ESM.docx]

| **Supplemental Table 1.** Treatment Details and Chemotherapies Used in the Study | |
| --- | --- |
| **Induction chemotherapy (n)** |  |
| carboplatin+taxol | 54 |
| carboplatin+taxotere | 26 |
| cisplatin+taxotere | 24 |
| carboplatin+gemcitabine | 11 |
| cisplatin+taxotere+bevacizumab | 8 |
| cisplatin+gemcitabine | 6 |
| cisplatin+taxotere+erlotinib | 4 |
| cisplatin+carboplatin+taxotere | 3 |
| cisplatin+gemcitabine+taxotere | 3 |
| carboplatin+pemetrexed | 1 |
| cisplatin+taxol | 1 |
| carboplatin+VP16 | 1 |
| cisplatin+taxol+erlotinib | 1 |
| cisplatin+mitomycin+vinblastine | 1 |
| carboplatin+taxol+erlotinib | 1 |
| carboplatin+taxotere+erlotinib | 1 |
| carboplatin+mitomycin | 1 |
| cisplatin+navelbine | 1 |
| taxotere | 1 |
| erlotinib | 1 |
| cisplatin+taxotere+pemetrexed+erlotinib+bevacizumab | 1 |
| unknown | 1 |
| **Adjuvant Chemotherapy (n)** |  |
| none | 131 |
| carboplatin+taxol | 7 |
| bevacizumab | 6 |
| erlotinib | 4 |
| taxotere | 1 |
| carboplatin+gemcitabine | 1 |
| gemcitabine+navelbine+taxotere+pemetrexed+bevacizumab | 1 |
| gemcitabine+navelbine+bevacizumab+WT1 vaccine x 10 | 1 |
| unknown | 1 |

| **Supplemental Table 2.** Sites of Any Failure | |
| --- | --- |
| Sites of Failure | n (%) |
| No relapse | 78 (51.0%) |
|  |  |
| Locoregional (n) | 38 (24.8%) |
| Mediastinal Nodes | 35 |
| Hilar Nodes | 9 |
| Surgical Site | 5 |
| Supraclavicular Nodes | 3 |
|  |  |
| Distant (n) | 59 (38.6%) |
| Brain | 25 |
| Lung | 13 |
| Bone | 7 |
| Adrenal | 5 |
| Liver | 3 |
| Skin | 2 |
| Distant Lymph Nodes | 2 |
